# Concise Review: Functional Roles and Therapeutic Potentials of Long Non-coding RNAs in Cholangiopathies

**DOI:** 10.3389/fmed.2020.00048

**Published:** 2020-02-20

**Authors:** Keisaku Sato, Shannon Glaser, Heather Francis, Gianfranco Alpini

**Affiliations:** ^1^Division of Gastroenterology and Hepatology, Department of Medicine, Indiana University School of Medicine, Indianapolis, IN, United States; ^2^Department of Medical Physiology, Texas A&M University, College of Medicine, Bryan, TX, United States; ^3^Richard L. Roudebush VA Medical Center, Indianapolis, IN, United States

**Keywords:** cholangiocytes, bile duct, microRNAs, long non-coding RNAs, cholangiocarcinoma

## Abstract

Long non-coding RNAs (lncRNAs) are RNAs with lengths exceeding 200 nucleotides that are not translated into proteins. It is well-known that small non-coding RNAs, such as microRNAs (miRNAs), regulate gene expression and play an important role in cholangiopathies. Recent studies have demonstrated that lncRNAs may also play a key role in the pathophysiology of cholangiopathies. Patients with cholangiopathies often develop cholangiocarcinoma (CCA), which is cholangiocyte-derived cancer, in the later stage. Cholangiocytes are a primary target of therapies for cholangiopathies and CCA development. Previous studies have demonstrated that expression levels of lncRNAs are altered in the liver of cholangiopathies or CCA tissues. Some lncRNAs regulate gene expression by inhibiting functions of miRNAs leading to diseased liver conditions or CCA progression, suggesting that lncRNAs could be a novel therapeutic target for those disorders. This review summarizes current understandings of functional roles of lncRNAs in cholangiopathies and seek their potentials for novel therapies.

## Introduction

It has been well-known since early studies that the human genome contains very small percentage (~1%) of exons of protein-coding genes (1). Although ~5-10% of the human genome is transcribed into RNAs, the large portions of RNA sequences do not code functional proteins (2). In recent years, these non-coding RNAs have been classified according to their lengths and characteristics, and especially small non-coding RNAs called microRNAs (miRNAs) have been studied to understand the pathophysiology of human diseases (3). Altered expression levels of miRNAs are a hallmark in diseased conditions, and the regulation of gene expression by miRNAs plays a critical role in pathogenesis of various human disorders including liver diseases (4). miRNAs could be useful as biomarkers to diagnose liver diseases including liver fibrosis and cancer, and could be a novel therapeutic target to regulate specific gene expression as well as cell events (5). Long non-coding RNAs (lncRNAs) are another class of non-coding RNAs that are >200 bp long. While the major function of miRNAs is to target mRNAs and regulate their expressions, various functions of lncRNAs have been suggested including regulation of gene expression, X-chromosome inactivation, telomere regulation, and chromatin structure regulation (3). Although functions of large numbers of lncRNAs are undefined, they could play a key role in the pathophysiology of liver diseases as well as miRNAs.

Cholangiopathies include bile duct disorders, such as primary sclerosing cholangitis (PSC), primary biliary cholangitis (PBC), and biliary atresia, which are characterized by a syndrome of biliary obstruction resulting from infection-related inflammation or autoimmune responses (6–8). Numbers of miRNAs have been identified in patients with cholangiopathies representing their potentials as novel diagnostic biomarkers or therapeutic targets (9–11). Recent, studies have also demonstrated that lncRNAs may be associated with pathogenesis and diseased conditions during cholestatic liver injury and could be another therapeutic target for cholangiopathies. This review summarizes current understandings of functional roles of lncRNAs and their potentials as therapeutic targets in cholangiopathies.

## Long Non-Coding RNAs IN Cholangiopathies

### Cholestatic Liver Injury and Primary Sclerosing Cholangitis

#### MEG3

Previous studies suggested the association of lncRNA maternally expressed gene 3 (MEG3) with liver fibrosis and hepatocellular carcinoma (12, 13). Another study has demonstrated that MEG3 interacts with RNA-binding protein polypyrimidine tract-binding protein 1 (PTBP1), which binds to small heterodimer partner (SHP) (14). SHP is a key regulator for bile acid synthesis by regulating cytochrome P450 family 7 subfamily A member 1 (Cyp7a1) and cytochrome P450 family 8 subfamily B member 1 (Cyp8b1), which are enzymes for bile acid synthesis from cholesterol (15). The PTBP1-MEG3 complex destabilizes SHP mRNA leading to its degradation and elevated Cyp7a1 and Cyp8b1 expression. Overexpression of MEG3 induced SHP degradation and elevated bile acid synthesis resulting in cholestatic liver injury in mice (14). These findings suggest that MEG3 is associated with pathogenesis of cholestatic liver injury and could be a therapeutic target to manage bile acid homeostasis and improve liver conditions.

#### H19

Zhang et al. have demonstrated that B-cell lymphoma protein 2 (Bcl2) is a key regulator of bile acid homeostasis, and overexpression of Bcl2 increases serum levels of bile acids leading to cholestatic liver injury in mice (16). Overexpression of Bcl2 induced SHP protein degradation as well as upregulation of lncRNA H19 (16). This study has demonstrated that SHP is a transcriptional repressor of H19, and overexpression of SHP and knockdown of H19 attenuated Bcl2-induced cholestatic liver injury *in vivo*, suggesting the association of H19 with SHP expression and cholestatic liver diseases (16). Bile duct ligation (BDL) is a surgical obstruction of common bile duct performed in rodents, which is utilized as an animal model of cholestatic liver injury (17). Song et al. have demonstrated that H19 expression is elevated in the liver after BDL, and overexpression of H19 exacerbates BDL-induced liver damage and fibrosis in mice (18). H19 deficient mice represented attenuated liver damage and fibrosis compared to wild-type mice after BDL, indicating the association of expression levels of H19 and liver conditions during cholestatic liver injury (18). Multidrug resistance 2 knockout (*Mdr2*^−/−^) mice are the most common transgenic mice that are utilized as the animal model of human PSC (19). *Mdr2*^−/−^ mice represent liver damage and fibrosis as well as elevated H19 expression in the liver, especially in female mice (20). Downregulation of H19 attenuated liver damage and fibrosis in *Mdr2*^−/−^ mice, suggesting that H19 could be a therapeutic target for the management of liver conditions in PSC (20).

### H19 Carried in Extracellular Vesicles

Exosomes and microparticles are extracellular vesicles (EVs) that are secreted from cells. Exosomes are small EVs (~100 nm in diameter) formed and secreted through the endosomal network, and microparticles (0.1–1 μm) are larger EVs formed by outward budding of the plasma membrane (21). These membrane-bound vesicles contain cargo mediators including DNAs, RNAs, and proteins, and secreted EVs from donor cells can be transferred into recipient cells delivering those cargo mediators (22, 23). This EV-mediated cell-to-cell communication followed by the regulation of cellular events plays a key role in the pathophysiology of liver diseases. A previous study has demonstrated that expression levels of H19 are elevated in the liver of PSC patients as well as in the mouse livers after carbon tetrachloride (CCl_4_)-induced liver damage, and CCl_4_ administration also increases levels of H19 carried in EVs isolated from mouse serum (24). H19-enriched cholangiocyte-derived EVs decreased SHP expression in hepatocytes, and injection of serum EVs isolated from *Mdr2*^−/−^ mice increased bile acid synthesis and exacerbated liver conditions in other *Mdr2*^−/−^ mice, suggesting EV-mediated cell-to-cell communication via cargo H19 (24). Since patients with liver cirrhosis have serum EVs carrying elevated levels of H19 compared to those from healthy individuals, H19-carrying EVs may play a critical role in the pathogenesis of cholestatic liver diseases and liver cirrhosis (24). Another study has demonstrated the correlation between expression levels of H19 and fibrogenic markers including collagen I and alpha smooth muscle actin (αSMA) in patients with PSC and PBC as well as in BDL and *Mdr2*^−/−^ mouse models (25). H19-enriched cholangiocyte-derived EVs induced proliferation and activation of hepatic stellate cells (HSCs) leading to fibrogenesis and cholestatic liver fibrosis *in vivo* (25). These studies suggest that EVs and cargo H19 delivery from cholangiocytes to other liver cells such as hepatocytes and HSCs are a critical step for pathogenesis of cholestatic liver injury, and H19 could be another therapeutic target for the treatment of liver fibrosis.

### Primary Biliary Cholangitis

PBC is an autoimmune disorder which is characterized by bile duct obstruction and cholestasis caused by intrahepatic bile duct destruction and inflammation (26). The cause of autoimmunity against bile ducts and cholangiocytes is still unknown. Therefore, previous studies have performed genotyping and association studies to identify susceptible loci or genes. A previous study has performed fine-mapping and association studies using a cohort of 2,861 cases and have identified three candidate loci that are associated with PBC (27). Hrdlichova et al. have extracted RNAs from seven immune cell types (granulocytes, monocytes, NK cells, B cells, memory T cells, naïve CD4+ and naïve CD8+ T cells) to obtain RNA sequencing libraries for patients with autoimmune disorders including PBC (28). This study has demonstrated that various lncRNAs expressed in immune cells are shared between autoimmune disorders, and NK cells, memory T cells and CD8+ cells in PBC patients have enriched those shared lncRNAs (28). Although this study suggests that lncRNAs may contribute to autoimmunity and pathogenesis of PBC, current studies are limited and detailed mechanisms and functional roles of lncRNAs in PBC are largely unknown.

### Biliary Atresia

Biliary atresia is a progressive bile duct disorder in infants representing cholestasis, jaundice, and liver fibrosis (29). Although previous studies has suggested the association between perinatal viral infection and biliary atresia development in infants, detailed mechanisms of pathogenesis in biliary atresia are still undefined (30). Chen et al. have performed genome-wide association study using a cohort of 343 non-related biliary atresia patients and 1,716 healthy controls to identify susceptible loci to biliary atresia (31). This study identified numbers of candidate loci, and one of significant SNPs was located in the gene *ADD3-AS1*, which encodes an lncRNA (31). Pseudogenes are DNA sequences that are related to genes but do not encode fully functional proteins. Therefore, transcripts of pseudogenes are recognized as lncRNAs. Pseudogenes and pseudogene-derived lncRNAs can be functional by regulating gene expression and could be a therapeutic target (32, 33). Annexin A2 (ANXA2) pseudogene 3 (ANXA2P3) is a pseudogene related to ANXA2. Previous studies have demonstrated that upregulation of ANXA2 is associated with liver fibrosis and can be useful as a biomarker for hepatitis B virus-related liver fibrosis (34, 35). Expression levels of ANXA2 as well as ANXA2P3 are also upregulated in liver tissues of biliary atresia patients, indicating that ANXA2P3 may be involved in the pathophysiology of biliary atresia development (36). As mentioned previously, lncRNA H19 is upregulated in the liver of patients with PSC and mouse models of PSC (24, 25). Another study analyzed H19 expression levels in biliary atresia patients and found that H19 was upregulated in the liver of biliary atresia patients compared to healthy individuals, and the expression of H19 was correlated with the expression of fibrogenic markers αSMA and transforming growth factor beta 1 (TGF-β1) (37). This study has demonstrated that H19 regulates functions of miRNAs let-7 families by binding them leading to elevated expression of the target of let-7, high-mobility group AT-hook 2 (HMGA2) (37). Decreased levels of let-7 are associated with ductular reaction and liver fibrosis during cholestatic liver injury (38). These studies suggest that lncRNAs are associated with PBC and biliary atresia although further studies are required to elucidate detailed mechanisms.

## Cholangiocarcinoma

### lncRNAs as Competing Endogenous RNAs in CCA

Cholangiocarcinoma (CCA) is a cancer that is derived from the biliary tree, and patients with PSC have a high risk for the development of CCA (39). Functions of lncRNAs have attracted interests in recent CCA studies because accumulating evidence suggests that lncRNAs may play a key role in cancer development, proliferation, and invasion of CCA. H19 binds to let-7 families and inhibit their functions like an let-7 sponge, as mentioned (37). lncRNAs function as competing endogenous RNAs (ceRNAs), which interrupt miRNA functions and alter protein expression, and this may be a characteristic hallmark in CCA. Genome-wide data analysis or RNA-Seq profiling identified various lncRNAs and ceRNA networks associated with CCA, and some candidate lncRNAs are significantly associated with survival rates (40–42). Recent studies have identified a number of lncRNAs that are associated with CCA progression and invasion. This review introduces selected studies of lncRNAs in CCA especially from recent studies. For other lncRNAs in CCA, see previous schematic reviews (43, 44).

### Functional Roles of lncRNAs in CCA

Previous studies have demonstrated that expression levels of lncRNA H19 are elevated in PSC and biliary atresia patients as described previously (24, 37). A study using tissue samples from patients with perihilar, distal, or intrahepatic CCA (iCCA) has represented that H19 expression is upregulated in CCA tissues compared to corresponding non-tumor tissues, and expression levels of H19 are associated with poor survival rates of patients (45). This study also demonstrated that H19 induced cell proliferation and migration in CCA cell lines RBE and QBC939 cells (45). Wang et al. analyzed lncRNA profiles expressed in CCA cell lines, RBE, QBC939, and SK-cha-1 cells, and found that lncRNAs H19 and HULC were upregulated during hydrogen peroxidase-induced oxidative stress (46). This study has demonstrated that H19 disrupts functions of let-7a and let-7b, which inhibit interleukin-6 (IL-6) expression as a target, and HULK interferes miR-372 and miR-373 that target C-X-C motif chemokine receptor 4 (CXCR4) (46). Since IL-6 and CXCR4 are associated with proliferation, migration, and metastasis of CCA (47–49), upregulation of H19 and HULC may lead to aberrant expression of IL-6 and CXCR4 as well as poor survival rates of CCA patients although further studies are required (46). Microarray analysis for lncRNAs using samples of fifty two CCA patients has identified five candidate lncRNAs that are significantly upregulated in iCCA tissues compared to adjacent non-tumorous tissues (50). Expression levels of one of those candidate lncRNAs, SNHG3, represented correlation with TNM stages, and patients with high SNHG3 expression had lower survival rates compared with patients with low SNHG3 expression (50). Another study using sixty CCA patients (intrahepatic, extrahepatic, and perihilar) has identified lnc-PKD2-2-3 as a candidate lncRNA, and high lnc-PKD2-2-3 expression was correlated with poor survival rates and high TNM stages (51). Although functional roles and targets of SNHG3 and lnc-PKD2-2-3 are undefined, these studies indicate the correlation between lncRNAs and CCA prognosis, and these lncRNAs could be utilized as a diagnostic biomarker for CCA. Epithelial-mesenchymal transition (EMT) is a process that epithelial cells adopt structural and functional characteristics of mesenchymal cells and is an important phenomenon in carcinogenesis and metastases in cancers including CCA (52, 53). Previous report have demonstrated that lncRNA-NEF and runt-related transcription factor 1 (RUNX1) are associated with EMT in cancer (54, 55). Liang et al. analyzed expression levels of lncRNA-NEF and RUNX1 in 56 iCCA patients and 42 healthy individuals and found that lncRNA-NEF was downregulated and RUNX1 was upregulated in iCCA tissues (56). This study has demonstrated that low expression levels of lncRNA-NEF are associated with poor survival rates, and lncRNA-NEF expression is negatively correlated with RUNX1 expression in iCCA patients (56). FENDRR is a lncRNA, which is downregulated in various cancers such as breast cancer, prostate cancer, and hepatocellular carcinoma (57–59). A study using 60 CCA patients has found that expression of FENDRR is downregulated in CCA tissues compared to non-cancerous tissues, and FENDRR expression is negatively correlated with expression of survivin (60). Survivin is a protein that inhibits apoptosis and upregulated in cancers (61, 62). FENDRR repressed proliferation, migration, and invasion of CCA cell lines HuCCT1 and QBC939 cells via regulation of survivin (60). An *in vitro* study using CCA cell lines (HuCCT1, Huh-28, KKU-214, and RBE) has demonstrated that CCA cells express elevated levels of lncRNA LINC01061 (63). LINC01061 binds to miR-612 and inhibits functions of miR-612, which targets semaphoring-4D (SEMA4D) (63). Since SEMA4D promotes invasion and metastasis of cancers (64, 65), this study indicates that LINC01061 functions as ceRNA for SEMA4D by sponging miR-612 leading to cell proliferation and migration of CCA cell lines (63). These studies suggest that expression levels of lncRNAs are associated with cell proliferation, migration, and invasion of CCA, and lncRNAs play an important role in physiological events of CCA cells by regulating protein expression. Table S1 summarizes lncRNAs identified in cholangiopathies and CCA.

## Candidate Therapeutic Approaches FOR lncRNAs

Current studies represent the association of lncRNAs with cholangiopathies and abnormal liver functions, such as excess bile acid synthesis and liver fibrosis as well as CCA characteristics, such as CCA cell migration and invasion, metastasis, or prognosis. These findings suggest that lncRNAs could be a novel therapeutic target to manage disease conditions in cholangiopathies.

### RNA Interference Targeting lncRNA

The majority of lncRNAs associated with cholangiopathies is upregulated in the diseased liver. RNA interference technology using shRNA or siRNA can be utilized to manage liver conditions. For example, shRNA targeting LINC01061 decreased cell proliferation and increased apoptosis in CCA cell lines KKU-214 and RBE cells (63). Antisense oligonucleotides that inhibit lncRNA functions or induce lncRNA degradation by RNaseH can be utilized for lncRNA silencing. Treatments of antisense oligonucleotides for lncRNA MALAT1 decrease tumor volumes and metastases in the mouse model of lung cancer (66). Gene knockout targeting lncRNAs is another approach for cholangiopathies. H19 is upregulated during cholestatic liver injury, and H19^−/−^ mice represent attenuated liver fibrosis during BDL compared to wild-type mice (25). Previous studies have introduced a technique for lncRNA silencing using zinc finger nucleases to induce lncRNA destabilization and degradation leading to 1,000-fold decreased expression of MALAT1 (66, 67). However, current studies are limited for cholangiopathies and CCA, and the majority of current studies using RNA interference is based on *in vitro* experiments. Further studies are required to establish the methodology for effective lncRNA silencing *in vivo*.

### Induction of lncRNA Expression

Some lncRNAs could be therapeutic or protective against liver diseases or cancer. For example, expression levels of lncRNA-NEF and FENDRR are downregulated in CCA tissues compared to normal tissues (54, 60). Overexpression of these lncRNAs inhibited cell migration and invasion of CCA cell lines HuCCT1, QBC939, or TFK-1 cells, indicating the potentials of lncRNA induction as another therapeutic approach for CCA (54, 60). As well as lncRNA silencing, lncRNA induction has same limitations: (i) Current studies are limited in the use of *in vitro* cultured CCA cell lines; and (ii) Technical difficulties to induce specific lncRNAs expression in specific cell types such as CCA cells. Gene therapy using a plasmid encoding the target gene has been performed for breast cancer (68), and the methodology could be modified to target therapeutic/protective lncRNAs in cholangiopathies although further studies are needed to seek their potentials.

### Small Molecule Inhibitors

Functions of lncRNAs could be impaired by small molecules. For example, some lncRNAs function as ceRNA by sponging miRNAs and regulating protein expression. Administration of small molecules that bind to the region for miRNA sponging may inhibit interaction between miRNAs and lncRNAs leading to effective inhibition of the target protein expression by miRNAs. Some lncRNAs interact with proteins to form a complex, and this lncRNA-protein complex function as an inhibitor that suppresses expression of the specific proteins. For example, lncRNA MEG3 interacts with PTBP1 to form a complex. This PTBP1-MEG3 complex binds to and destabilizes mRNA of SHP leading its degradation followed by elevated bile acid synthesis and cholestatic liver injury (14). Small molecules that interfere RNA-protein interaction between MEG3 and PTBP1 may have therapeutic effects for cholestatic liver injury induced by downregulated SHP and aberrant bile acid synthesis. Small molecules that bind to the specific region of lncRNAs and inhibit its correct folding could be utilized to induce lncRNA degradation and functional inhibition. Although these ideas may be theoretically possible, studies are still ongoing and no candidate molecules for cholangiopathies to be utilized for clinical trials are available to date.

### Targeting or Utilization of EVs

Recent studies have demonstrated that EVs play a key role in cholangiopathies. H19 is upregulated in PSC patients, and cholangiocyte-derived EVs transfer cargo H19 to hepatocytes or HSCs in diseased conditions leading to bile acid synthesis or fibrogenesis, respectively (24, 25). Drugs that decrease EV production or secretion may inhibit fibrogenic cell-to-cell communication via H19-enriched EVs in PSC. High throughput screen assay has identified compounds that modulate EV biogenesis or release in prostate cancer cells (69). These compounds could also be effective on EV production or secretion in cholangiocytes or CCA cells leading to improved liver conditions although further studies are required.

EVs functions as a disease-inducing mediator carrier during cholestatic liver injury by delivering H19 from cholangiocytes to other liver cells (24, 25). This means that EVs could be utilized as a drug or therapeutic mediator carrier to manage liver conditions. A recent study has demonstrated that injection of EVs isolated from liver stem cells attenuates ductular reaction and liver fibrosis in *Mdr2*^−/−^ mice via delivering cargo let-7, indicating the potentials of EVs as a therapeutic tool and an miRNA carrier (19). Injection of EVs carrying mediators, such as small molecules or nucleotides targeting lncRNAs could be performed to regulate lncRNA functions *in vivo* and manage liver conditions. EVs carrying candidate mediators such as miRNAs can be produced by cell transfection (70), and previous studies have also reported that modification of EV cargo mediators for miRNAs or miRNA inhibitors can be accomplished by electroporation (71, 72). Although further studies are required, these studies indicate that the methodology could be modified for lncRNAs or mediators targeting lncRNAs that are carried in EVs, and lncRNAs-targeting EVs could be useful to manage liver conditions and cancer progression.

## Conclusion

**Figure 1:**
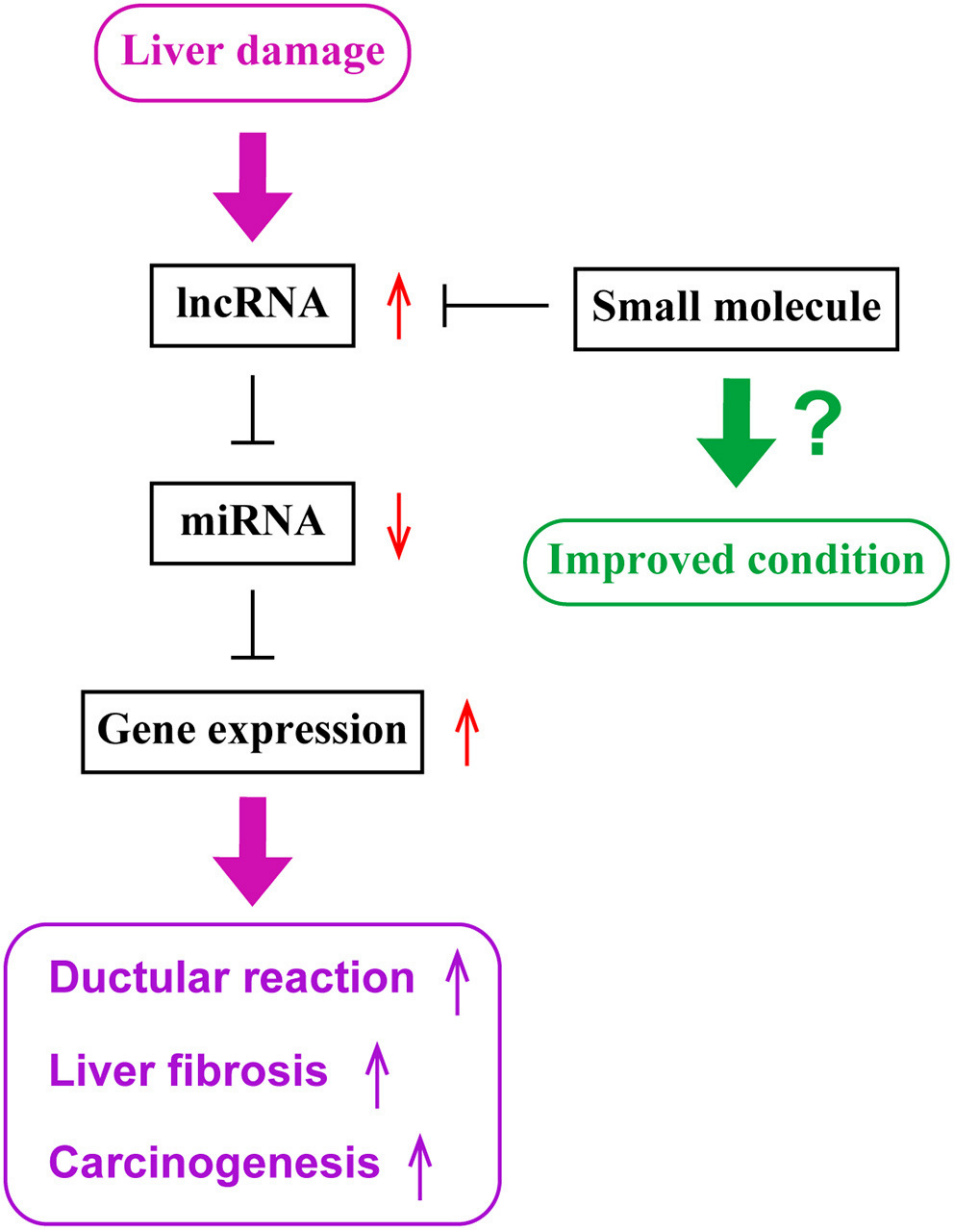
The role of lncRNAs in liver diseases. During liver damage, expression levels of long non-coding RNAs (lncRNAs), such as H19, are elevated in the liver. These lncRNAs sponge microRNAs (miRNAs), such as let-7 families, and inhibit their functions. Since miRNAs inhibit the expression of target genes, such as HMGA2, elevated levels of lncRNAs lead to enhanced gene expressions of target genes. Elevated gene expression is associated with ductular reaction, liver fibrogenesis and inflammation, or carcinogenesis or tumor progression. Small molecules targeting lncRNAs may be utilized as novel therapeutic tools to inhibit lncRNA functions and maintain liver homeostasis.

Current studies have demonstrated that expression levels of lncRNAs are associated with diseased conditions of cholestatic liver diseases and CCA. lncRNAs function as ceRNAs by sponging miRNAs to regulate protein expression. Although there are various approaches available that are theoretically possible to regulate functions of lncRNAs leading to the management of cholangiopathies, further studies are required to understand detailed mechanisms of functions of lncRNAs and to develop the methodology for a novel therapy targeting lncRNAs. Figure 1 represents a diagram for the roles of lncRNAs in liver diseases.

## Author Contributions

KS designed the study and wrote the manuscript. SG and HF critically reviewed the manuscript. GA conducted and designed the project, and critically reviewed the manuscript.

### Conflict of Interest

The authors declare that the research was conducted in the absence of any commercial or financial relationships that could be construed as a potential conflict of interest.

## Abbreviations

ANXA2: Annexin A2
ANXA2P3: Annexin A2 pseudogene 3
αSMA: alpha smooth muscle actin
Bcl2: B-cell lymphoma protein 2
BDL: bile duct ligation
CCl_4_: carbon tetrachloride
ceRNAs: competing endogenous RNAs
Cyp7a1: cytochrome P450 family 7 subfamily A member 1
Cyp8b1: cytochrome P450 family 8 subfamily B member 1
CXCR4: C-X-C motif chemokine receptor 4
EMT: epithelial-mesenchymal transition
HMGA2: high-mobility group AT-hook 2
HSCs: hepatic stellate cells
iCCA: intrahepatic CCA
IL-6: interleukin-6
long non-coding RNAs: lncRNAs
Mdr2: multidrug resistance 2
MEG3: maternally expressed gene 3
miRNAs: microRNAs
PBC: primary biliary cholangitis
PSC: primary sclerosing cholangitis
PTB1: polypyrimidine tract-binding protein 1
RUNX1: runt-related transcription factor 1
SHP: small heterodimer partner
SEMA4D: semaphoring-4D
TGF-β1: transforming growth factor beta 1.
