# Supplementary material for: Concise Review: Functional Roles and Therapeutic Potentials of Long Non-coding RNAs in Cholangiopathies
Source: Front Med (Lausanne). 2020 Feb 20;7:48. doi: 10.3389/fmed.2020.00048 (PMC7045865; doi:10.3389/fmed.2020.00048)
Supplement: Supplementary file 1 [file Data_Sheet_1.PDF]

Table 1. Selected lncRNAs identified in cholangiopathies and CCA.

lncRNA	Disease/injury/source	Upregulated/downregulated in diseased conditions	Targets/associated proteins	Functions
MEG3 (14)	Cholestatic liver injury	Upregulated	PTBP1, SHP	Induces bile acid synthesis and cholestatic liver damage
H19 (24)	PSC	Upregulated	SHP	Induces bile acid synthesis
ANXA2P3 (36)	BA	Upregulated	ANXA2	Unknown
H19 (37)	BA	Upregulated	let-7, HMGA2	Induces liver fibrosis
H19 (45, 46)	CCA tissues and cell lines	Upregulated	let-7a, let-7b, IL-6	Associated with poor prognosis

HULK (46)	CCA cell lines	Upregulated	miR-372, miR-373, CXCR4	Induces migration and invasion of CCA cells
SNHG3 (50)	iCCA tissues	Upregulated	Unknown	Associated with poor prognosis
lnc-PKD2-2-3 (51)	CCA tissues	Upregulated	Unknown	Associated with poor prognosis
lncRNA-NEF (56)	iCCA tissues	Downregulated	RUNX1	Inhibits cell migration and invasion
FENDRR (60)	CCA tissues, cell lines	Downregulated	Survivin	Inhibits cell migration and invasion

LINC01061 (63)	CCA cell lines	Upregulated	miR-612, SEMA4D	Induces cell proliferation and migration
